# Isolation and Characterization of a Process Impurity in Tizanidine Hydrochloride

**DOI:** 10.4103/0250-474X.70484

**Published:** 2010

**Authors:** E. K. S. Vijayakumar, V. G. Gore, A. Mahajan, M. Kumar

**Affiliations:** Mylan India Pvt. Ltd., Plot 1A/2, M.I.D.C. Industrial Estate, Taloja - 410 208, India; 1 USV Limited, Govandi, Mumbai-400 088, India; 2 Astrix Laboratories Ltd., Gaddapotharam, Kazipally Industrial Area, Medak Dist-502319, Andhra Pradesh, India

**Keywords:** Tizanidine, process impurity, MS, NMR, preparative HPLC

## Abstract

A new process impurity was detected during the HPLC analysis of Tizanidine hydrochloride (I) batches. The impurity (II) was isolated by preparative HPLC and characterized by NMR and Mass spectral analysis as 5-S-ethyl-N-(4,5-dihydro-1H-imidazol-2-yl)-2,1,3-benzothiadiazol-4-amine hydrochloride.

Tizanidine hydrochloride (I), chemically known as 5-chloro-N-(4,5-dihydro-1H-imidazol-2-yl)-2,1,3-benzothiadiazol-4-amine hydrochloride, is a centrally acting alpha2-adrenergic agonist and is used to treat the spasms, cramping and tightness of muscles caused by medical problems such as multiple sclerosis, spastic diplegia, back pain or certain other injuries of the spine or central nervous system[1–6].

During the HPLC analysis for related substances of tizanidine hydrochloride (I) batches, synthesized as per the route reported by Neumann[7], an unknown process impurity (II) was detected. The content of this impurity was found to be in the order of 0.05 % to 0.15 % w/w. The isolation and characterization of this unknown process impurity (II) was, therefore, necessary not only to meet the stringent regulatory requirement, but also to get an insight into the possible route of formation of the impurity. In addition to three related impurities *viz*. 4-amino-5-chloro-2,1,3-benzothiadiazole (related compound A), N-acetyltizanidine (related compound B) and 1-acetylimidazolodine-2-thione (related compound C) listed in USP monograph[8] on tizanidine hydrochloride (I), six process impurities of tizanidine hydrochloride (I) namely N-(4,5-dihydro-1H-imidazol-2-yl)-2,1,3-benzothiadiazole-4-amine hydrochloride, N-(5-chloro-2, 1,3-benzothiadi-azol-4-yl) thiourea, dimer of tizanidine HCl, S-methyl-N-(5-chloro-2, 1,3-benzothiadiazol-4-yl) isothiouronium iodide, 4-amino-5-chloro-2, 1,3-benzothiadiazole and N,N-bis(5-chloro-2, 1,3-benzothiadiazol-4-yl)-N-(4,5-dihydro-1H-imidazol-2-yl) guanidine were reported[9]. Spectral data of II did not corroborate with the structures of these impurities suggesting that II is hitherto an unreported impurity. This paper describes the isolation by preparative HPLC and characterization of the unknown process impurity (II) by spectral analysis.

Sample of tizanidine hydrochloride (I) was synthesized and characterized in Mylan India Pvt. Ltd (Formerly Merck Development Centre Pvt. Ltd.), India. Sodium dihydrogen phosphate, methanol (HPLC grade) and phosphoric acid were procured from Merck India Ltd., Mumbai, India. The chromatographic purification was performed on a Nova Prep 200 (Merck Hitachi) preparative HPLC system consisting of L-7400 UV detector and HSM software and a built-in autosampler for fraction collection. The purity of the fractions was checked on a Merck Hitachi HPLC system consisting of L-7100 pump, L-7300 Column oven, L-7200 Autosampler, L-7420 detector and HSM data acquisition software. The mass spectra were obtained on a Applied Biosystems API 4000 triple quadrupole spectrometer using electrospray ionization in positive mode. HR-MS spectrum was obtained on a Micromass Q-TOF micro spectrometer using electrospray ionization in positive mode. NMR spectra were recorded on a Bruker AV 300 spectrometer.

The mobile phase A was a mixture of 0.02 M aqueous sodium dihydrogen phosphate, pH 3.0 with dilute orthophosphoric acid-methanol (90:10 v/v), while mobile phase B was 0.02 M aqueous sodium dihydrogen phosphate, pH 3.0 with dilute orthophosphoric acid-methanol (20:80 v/v). The gradient program used was min/%B: 10/0, 30/50, 31/0 and 40/0. The preparative HPLC column used was Waters C18 Symmetry (19×150 mm), 7 μ. The monitoring wavelength was 225 nm and the flow rate was 24 ml/min. A stock solution of 3% w/v tizanidine hydrochloride (I) was prepared in water for the isolation of process impurity (II) and 4 ml was injected per run. The fraction containing the enriched impurity (II) was re-chromatographed using a C18 Symmetry (7.8×150 mm), 7 μ. at a flow rate of 4.0 ml/min with an injection volume of 50 μl. Analytical HPLC was performed using the same conditions except that the column used was Symmetry C18 (150×3.9 mm), 5 μ with a flow rate of 1 ml/min for checking the purity.

The preparative HPLC fraction containing impurity was evaporated to dryness on a rotavapor at 30°under vacuum. The residue was suspended in minimum quantity of dry methanol, sonicated for 2 min and kept overnight in refrigerator. The suspension was filtered immediately under vacuum to remove the undissolved phosphates. The filtrate was concentrated on a rotavapor at 30°under vacuum to dryness to get impurity (II) as a yellow powder.

^1^H NMR spectra of I and II were recorded in DMSO-d6 and DMSO-*d*_6_ +2 drops of D_2_O at a concentration of approximately 5 mg/0.7 ml, while ^13^C NMR and DEPT-135 spectra in DMSO-*d*_6_ at 25 mg/0.7 ml. The chemical shifts were reported on δ scale in ppm relative to DMSO-*d*_6_ (δ_H_ 2.51 and δ_C_ 39.5).

The unknown process impurity (II) was obtained as a yellow powder having a purity of 94% by HPLC. The ESI-MS gave a molecular ion peak at *m/z* 280.3 (M+H)^+^. A comparison of NMR spectra (Table 1) of process impurity (II) with those of tizanidine hydrochloride (I) indicated the presence of additional signals [δ_H_: 3.18 (q, 2H) and 1.28 (t, J=7.3 Hz, 3H); δ_C_: 25.9 (CH_2_) and 14.6 (CH_3_)] in II, which were attributed to an ethyl group. The relatively downfield shift of methylene was due to its attachment to a hetero atom. The presence of only two aromatic methines [δ_H_: 8.09 (d, J=9.3 Hz, 1H) and 7.86 (d, J=9.3 Hz, 1H); δ_C_: 129.3 and 121.3] together with the absence of isotopic pattern for chlorine in the mass spectrum suggested that S-ethyl group was present in place of chlorine. During the conversion of tizanidine to the corresponding HCl salt, II was also expected to form a HCl salt, which was confirmed by silver nitrate test. Thus, the structure of impurity (II) was established as 5-S-ethyl-N-(4,5-dihydro-1H-imidazol-2-yl)-2, 1,3-benzothiadiazol-4-amine hydrochloride (fig. 1). The structure was further confirmed by comparison with synthetic sample prepared subsequently. The possible route for the formation of II was due to the displacement of chlorine atom by ethanethiol liberated during the conversion of precursor *viz*. S-ethyl-N-(5-chloro-2, 1,3-benzothiadiazole-4-yl) isothiouronium bromide (III) to tizanidine hydrochloride (I) using ethylenediamine/p-toluenesulfonic acid in toluene/water[7].

**TABLE 1:** NMR DATA OF TIZANIDINE HYDROCHLORIDE (I) AND IMPURITY (II)

Position	^13^C (δ)		^1^H (δ)	
	I	II	I	II
a	158.4	158.5	-	-
4	154.2	153.8	-	-
2	152.0	152.1	-	-
5	132.9	138.8	-	-
6	131.4	129.3	8.18 (d, J = 9.3 Hz, 1H)	8.09 (d, J = 9.3 Hz, 1H)
3	124.9	123.2	-	-
7	121.9	121.3	7.92 (d, J = 9.3 Hz, 1H)	7.86 (d, J = 9.3 Hz, 1H)
b, c	42.9	42.9	3.69 (s, 4H)	3.65 (bs, 4H)
S-Ethyl	-	25.9 (CH_2_)	-	3.18 (q, 2H)
		14.6 (CH_3_)	-	1.28 (t, J = 7.3 Hz, 3H)
NH	-	-	11.31 (bs, 1H)	10.99 (bs, 1H)
NH, HCl	-	-	8.56 (s, 2H)	8.46 (bs, 2H)

**Fig. 1:**
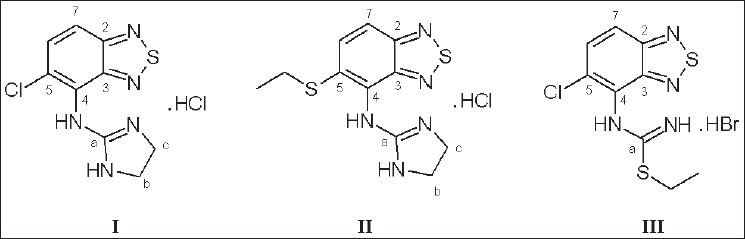
Chemical structures of tizanidine hydrochloride (I), impurity (II) and III I: 5-chloro-N-(4,5-dihydro-1H-imidazol-2-yl)-2, 1,3-benzothiadiazol-4-amine hydrochloride; II: 5-S-ethyl-N-(4,5-dihydro-1H-imidazol-2-yl)-2, 1,3-benzothiadiazol-4-amine hydrochloride; III: S-ethyl-N-(5-chloro-2, 1,3-benzothiadiazole-4-yl) isothiouronium bromide
